# A Novel Multi-Core Parallel Current Differential Sensing Approach for Tethered UAV Power Cable Break Detection

**DOI:** 10.3390/s25165112

**Published:** 2025-08-18

**Authors:** Ziqiao Chen, Zifeng Luo, Ziyan Wang, Zhou Huang, Yongkang He, Zhiheng Wen, Yuanjun Ding, Zhengwang Xu

**Affiliations:** 1 Detroit Green Technology Institute, Hubei University of Technology, Wuhan 430068, China; 18186653635@163.com; 2 School of Electrical and Electronic Engineering, Hubei University of Technology, Wuhan 430068, China; 15889891189@163.com (Z.L.); 13720162698@163.com (Z.W.); 13252293085@163.com (Y.H.); www_040521@163.com (Z.W.); 15527375587@163.com (Y.D.); xuzw72@hbut.edu.cn (Z.X.)

**Keywords:** current differential sensing, multi-core parallel, ACS712 sensor, tethered UAV, cable break detection

## Abstract

Tethered unmanned aerial vehicles (UAVs) operating in terrestrial environments face critical safety challenges from power cable breaks, yet existing solutions—including fiber optic sensing (cost > USD 20,000) and impedance analysis (35% payload increase)—suffer from high cost or heavy weight. This study proposes a dual innovation: a real-time break detection method and a low-cost multi-core parallel sensing system design based on ACS712 Hall sensors, achieving high detection accuracy (100% with zero false positives in tests). Unlike conventional techniques, the approach leverages current differential (*ΔI*) monitoring across parallel cores, triggering alarms when *ΔI* exceeds *I_rate_*/2 (e.g., 0.3 A for 0.6 A rated current), corresponding to a voltage deviation ≥ 110 mV (normal baseline ≤ 3 mV). The core innovation lies in the integrated sensing system design: by optimizing the parallel deployment of ACS712 sensors and LMV324-based differential circuits, the solution reduces hardware cost to USD 3 (99.99% lower than fiber optic systems), payload by 18%, and power consumption by 23% compared to traditional methods. Post-fault cable temperatures remain ≤56 °C, ensuring safety margins. The 4-core architecture enhances mean time between failures (MTBF) by 83% over traditional systems, establishing a new paradigm for low-cost, high-reliability sensing systems in terrestrial tethered UAV cable health monitoring. Preliminary theoretical analysis suggests potential extensibility to underwater scenarios with further environmental hardening.

## 1. Introduction

UAVs (Unmanned Aerial Vehicles) [1] are generally powered by lithium batteries. Due to the weight of the batteries, the capacity of the batteries installed in the UAV is limited, and they can generally only support the UAV to fly for about half an hour. Unlike battery-powered UAVs, tethered systems transmit energy via the cables, enabling continuous operation without the limitations of onboard energy storage. Tethered UAVs are widely used in long-endurance fields such as agriculture [2], communications [3,4,5,6,7], measurement [8,9,10,11], and disaster response [12,13,14,15]—primarily in terrestrial environments—where low cost and lightweight design are critical—addressing practical needs unmet by existing solutions. Many scholars have also carried out in-depth research on the applications of tethered UAVs. In addition to the aforementioned literature, references [16,17,18,19,20,21,22] focus on the stability of UAVs, while references [23,24,25] provide references on the power supply systems of UAVs.

This literature provides rich references and promotes the wide application of tethered UAVs. However, a literature search shows that there is still a technical gap in the application field of tethered UAVs. Except for the research in reference [21], which has a certain correlation with the problem of cable breaks, no literature providing solutions for cable break detection in tethered UAVs has been found.

Despite their advantages, tethered systems face critical challenges. The reliance on these cables introduces a systemic vulnerability: progressive core degradation due to mechanical fatigue, environmental exposure, and repeated bending during deployment. Moreover, cable breaks are generally difficult to detect on the ground before they occur. When the UAV flies to a higher position, the cable is most likely to break due to the tension of its own weight.

Existing approaches to mitigate cable failures primarily focus on mechanical reinforcement, such as thickening cables or integrating tensile materials [21]. However, these methods can only extend cable lifespan, and they have brought the side effect of increasing weight and stiffness, compromising UAV maneuverability and energy efficiency. Alternative solutions, including backup batteries or supercapacitors, introduce additional complexity, cost, and payload burdens, thereby reducing flight endurance and wind resistance.

On power supply cable fault diagnosis, numerous studies provide valuable contextual insights. For instance, references [26,27,28] employ UAV-mounted high-resolution cameras and/or thermal sensors to detect broken strands in overhead power lines. These methods can detect broken strands in power supply cables, but they are difficult to transplant to detect power cables of tethered UAVs.

Although Fiber Bragg Grating (FBG) sensing [29] and impedance analysis [30] can be modified for use with tethered UAVs, they encounter considerable challenges in this application. FBG requires sophisticated wavelength demodulation equipment (cost > USD 20,000) and meticulous sensor bonding (30+ min per point). Impedance analysis demands an analyzer and complex signal injection/detection circuits (~USD 10,000 total cost).

Hall effect sensors, leveraging the advantages of non-contact measurement, millisecond-level response speed, and low cost, have been widely applied in real-time current monitoring of small electronic devices (e.g., motor overload protection, battery charge-discharge management). However, systematic research on their architectural design for achieving high-reliability fault identification through current differential signals in distributed break detection of multi-core parallel cables remains lacking.

This study introduces a paradigm-shifting multi-core parallel current differential sensing architecture designed to address these gaps. The innovation resides in three core advancements, with a focus on the design of a low-cost Hall sensor-based multi-core parallel sensing system:

- Multi-core Parallel Optimized Cable Architecture: By distributing current across multi-cores in parallel, the system achieves an >83% improvement in MTBF compared to traditional configurations. This design enables fault detection through real-time monitoring of current differentials (*ΔI* > *I_rate_*/2), eliminating the need for auxiliary energy storage;
- Ultra-Low-Cost Analog Signal Processing: Leveraging off-the-shelf components—ACS712 Hall effect sensors (USD 0.63 per unit) and LMV324 operational amplifiers (USD 0.07 per unit)—the hardware solution totals just USD 3, representing a 1/3000 cost reduction compared to FBG systems [29]. The analog circuitry achieves sub-10 ms response times, 8 times faster than impedance-based methods [30];
- Lightweight Break Detection: The detection circuit with low voltage and small current is composed of ACS712 Hall sensors (Allegro MicroSystems, Worcester, MA, USA) [31] and LMV324 operational amplifiers (Texas Instruments Incorporated, Dallas, TX, USA) [32], completely eliminating the need for UAV backup power supplies, reducing the UAV load by 18%, and the detection circuit reduces power consumption by 23% compared with traditional schemes.

By integrating these innovations, this research establishes a new benchmark for cost-effective, lightweight cable health monitoring in tethered UAV systems, directly addressing the safety imperatives of long-endurance missions in sectors ranging from critical infrastructure inspection to emergency response. While this study focuses on terrestrial applications, the core sensing principle may hold potential for underwater tethered UAVs with specialized waterproofing, though this requires separate validation beyond the scope of this work.

The subsequent sections detail the system design methodology, experimental validation across 50 flight tests (demonstrating 100% detection accuracy in tests), thermal safety analysis (post-fault temperatures ≤ 56 °C), progressive break simulation experiments, and a comprehensive discussion.

## 2. System Design

This section first presents the structure of the multi-core parallel power supply system, serving as the cornerstone for the innovative cable break detection principle detailed hereinafter. Through rigorous theoretical analysis of system reliability—pioneeringly modeling the MTBF enhancement brought by parallel architectures—it provides critical design guidelines for high-reliability implementations. Specifically, by taking a 4-core cable power supply system as a case study, this section analyzes the distinct current variations between normal operation and post-break scenarios in the positive power supply wires.

### 2.1. Multi-Core Parallel Power Supply Architecture

Since the tethered cable is a relatively heavy load of UAV, it is usually selected to use a thinner cable to transmit high-voltage DC power to reduce weight, as described in references [23,24,25]. For the traditional scheme, referred to as 1P1N hereinafter, there is 1 positive core (P1) and 1 negative core (N1) in the power supply cable. A multi-core parallel cable, which has more cores in the cable, is used to transmit power in this paper instead. For example, the 4-core system, referred to as 2P2N hereinafter, has 2 positive cores (P1, P2) and 2 negative cores (N1, N2); the 6-core system, referred to as 3P3N hereinafter, has 3 positive cores (P1, P2, P3) and 3 negative cores (N1, N2, N3).

**Figure 1:**
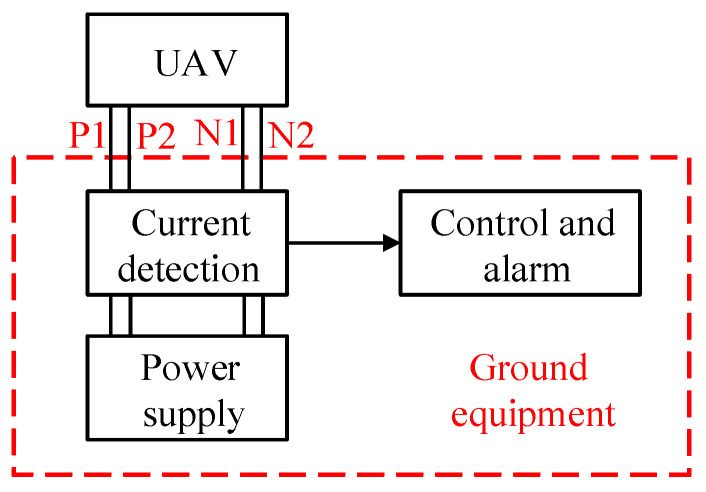
Structure of tethered UAV power supply system.

In the multi-core system, the power supply current of each core is detected for break detection. Let’s take the 2P2N system as an example. Each core is selected according to half of the rated load capacity. Then, the parallel connection of the two cores can provide the rated power supply capacity for the UAV. The weight increase is limited compared to a traditional 1P1N power supply cable. The structure of the entire tethered UAV power supply system is shown in Figure 1.

The ground equipment converts grid AC power to high-voltage DC power to power the UAV via the tethered cable. The UAV does not need to be equipped with batteries, supercapacitors, or other energy storage devices, but only an onboard power supply that can convert high-voltage DC power to the working voltage DC power for the UAV. The UAV also does not need to be modified to adapt to the detection circuit, which can reduce system complexity and deployment costs. The current detection part detects the current of each core in the cable. In the control and alarm part, the current detection result is used to determine whether there is a core break fault. If there is such a fault, an alarm signal is issued indicating that the UAV needs to be landed and the cable replaced.

### 2.2. System Reliability Analysis

Building on the multi-core parallel power supply architecture described in Section 2.1, this section explores the system reliability analysis. By examining the failure rates of parallel power cores, we derive MTBF for different configurations, providing a theoretical basis for the enhanced reliability of the proposed design.

Assume each power core (positive/negative) has an exponential failure distribution with constant failure rate *λ* (failures/hour). The system requires at least 1 positive core and 1 negative core to function normally.

For the traditional scheme (1P1N), the system fails if either P1 or N1 fails. The reliability function is:

(1) $$R_{1P1N}\left(t\right)\;=\;P\left({P1}_{normal}\right)\cdot P\left({N1}_{normal}\right)\;=\;e^{-\lambda t}\cdot e^{-\lambda t}=e^{-2\lambda t}$$

For the 4-Core System (2P2N), at least 1 of 2 positive cores and 1 of 2 negative cores must be normal. The possibility of normal positive and negative power:

(2) $$P\left(\geq 1\;positive\;normal\right)=1-(1-e^{-\lambda t}{)}^{2}$$

(3) $$P\left(\geq 1\;negative\;normal\right)=1-(1-e^{-\lambda t}{)}^{2}$$

System reliability:

(4) $$R_{2P2N}\left(t\right)=[1-{\left(1-e^{-\lambda t}\right)}^{2}{]}^{2}$$

For the 6-Core System (3P3N), at least 1 of 3 positive cores and 1 of 3 negative cores must be normal. The possibility of normal positive and negative power:

(5) $$P\left(\geq 1\;positive\;normal\right)=1-(1-e^{-\lambda t}{)}^{3}$$

(6) $$P\left(\geq 1\;negative\;normal\right)=1-(1-e^{-\lambda t}{)}^{3}$$

System reliability:

(7) $$R_{3P3N}\left(t\right)=[1-{\left(1-e^{-\lambda t}\right)}^{3}{]}^{2}$$

MTBF is the integral of the reliability function from *t* = 0 to *t* = ∞, thus:

(8) $${MTBF}_{1P1N}=\int_{0}^{\infty }R_{1P1N}\left(t\right)dt=\int_{0}^{\infty }e^{-2\lambda t}dt=\frac{1}{2\lambda }$$

(9) $$\begin{array}{cc} {MTBF}_{2P2N} & =\int_{0}^{\infty }R_{2P2N}\left(t\right)dt=\int_{0}^{\infty }{\left[1-{\left(1-e^{-\lambda t}\right)}^{2}\right]}^{2}dt\\ & =\int_{0}^{\infty }\left({4e}^{-2\lambda t}-4e^{-3\lambda t}+e^{-4\lambda t}\right)dt=\frac{4}{2\lambda }-\frac{4}{3\lambda }+\frac{1}{4\lambda }=\frac{11}{12\lambda } \end{array}$$

(10) $$\begin{array}{cc} {MTBF}_{3P3N} & =\int_{0}^{\infty }R_{3P3N}\left(t\right)dt=\int_{0}^{\infty }{\left[1-{\left(1-e^{-\lambda t}\right)}^{3}\right]}^{2}dt\\ & =\int_{0}^{\infty }\left({9e}^{-2\lambda t}-18e^{-3\lambda t}+{15e}^{-4\lambda t}-{6e}^{-5\lambda t}+e^{-6\lambda t}\right)dt\\ & =\frac{9}{2\lambda }-\frac{18}{3\lambda }+\frac{15}{4\lambda }-\frac{6}{5\lambda }+\frac{1}{6\lambda }=\frac{73}{60\lambda } \end{array}$$

The reliability comparative analysis are shown in Table 1.

The 4-core system enhances MTBF by 83% compared to the traditional scheme, while the 6-core system achieves a 143% improvement. The results demonstrate that multi-core parallel current differential sensing systems can improve system reliability significantly, making them suitable for high-requirement tethered UAV missions.

### 2.3. Break Detection Principle and Algorithm

If the tethered UAV is subjected to a huge external pulling force, all cables may break simultaneously. In this case, the UAV itself may have been damaged or directly fallen, so detecting whether the cables are broken is of little value. Even if the UAV is not problematic, detecting the complete break of cables while ensuring that the UAV does not fall requires enormous costs. Therefore, this paper does not consider such low-probability extreme scenarios and only focuses on cable break issues that occur during long-term normal use.

In the proposed multi-core power cable architecture, the probability of simultaneous cable breaks for all cores in the same polarity is negligible under normal operational conditions—unless subjected to extreme tensile forces. Specifically, progressive degradation from aging or repeated bending typically leads to stepwise core failure, where one core fails initially while others maintain current-carrying capability. This redundancy ensures the UAV remains powered, eliminating immediate crash risks.

Current sensing elements (e.g., Hall effect sensors or current transformers) convert per-core currents into proportional voltage signals with high fidelity. By comparing signals of the same polarity (positive/negative), the system detects abnormal current differentials: small *ΔI* indicates normal operation, while large discrepancies indicate partial or complete core breaks and trigger an alarm. This differential sensing mechanism leverages the parallel architecture to enable fault detection without compromising power continuity.

**Figure 2:**
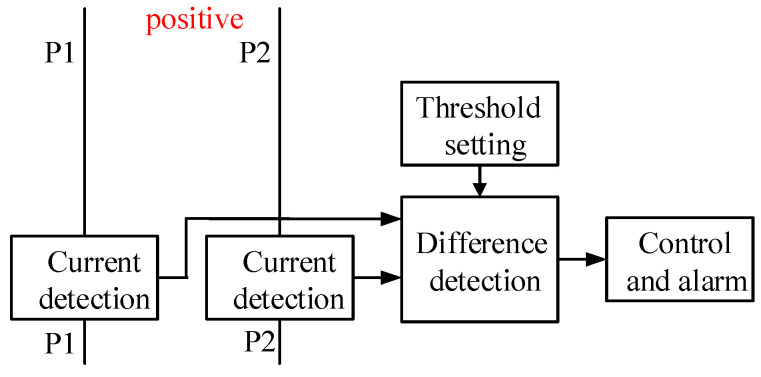
Positive end break detection principle.

Still using a 4-core cable as an example, two of the cores are used for each of the positive and negative ends of the power supply. All cores of the same polarity are directly paralleled, with one end connected to ground equipment and the other to the onboard power supply. The circuit principle for detecting a broken wire at the positive end is shown in Figure 2.

If the two positive cores are designated as P1 and P2, and the UAV’s rated operating current is *I_rate_*, each core should carry approximately *I_rate_*/2 under normal conditions as they are connected in parallel, resulting in a near-zero current differential. When P1 experiences a break, its current drops to zero while P2 assumes the full load *I_rate_*, yielding a differential of *I_rate_*. This stark contrast between faulty and healthy cores remains detectable even amid minor current imbalances or sensing errors. This inherent signal margin enables robust fault identification and timely alarm activation.

If *I*_1_ and *I*_2_ are used to represent the currents of P1 and P2, respectively, the cable break detection algorithm can be expressed as:

(11) $$\left\{\begin{array}{c} \Delta I\;=\;\left|I_{1}\;-\;I_{2}\right|\\ \Delta I\;>\;I_{threshold}\;=\;\frac{I_{rate}}{2} \end{array}\right.$$

where: *ΔI* is the current differential between the two cores, in A; *I_threshold_* is the threshold current, in A.

Consequently, the criterion for identifying core breaks is established by whether the current differential (*ΔI*) between the two parallel cores exceeds half of the rated current (*I_rate_*/2). Given that *ΔI* is bounded between 0 and *I_rate_*, and the tolerance interval of *I_rate_*/2 is symmetrically distributed in both positive and negative directions, this configuration demonstrates robust anti-interference capability.

## 3. Experiments and Results

This section commences with an introduction to the experimental subjects, namely the DJI (Dajiang Innovation Technology, Shenzhen, China) Phantom 4 UAV and the 4-core power cable. It details the current detection circuit (centering on ACS712 Hall sensors) and differential amplification circuit (based on LMV324 op-amps), analyzing their output changes under normal operation (no break) and fault conditions (break). A physical test platform was constructed, and fifty rounds of experimental trials were executed under both non-fault (intact cable) and fault (cable break) conditions to acquire corresponding test data. Finally, thermal characterization experiments were conducted to assess the potential overheating hazard of the cable following breaks. The experimental results demonstrate that the proposed system can accurately identify cable break faults, and no safety risks associated with overheating were observed.

### 3.1. Test Platform

**Figure 3:**
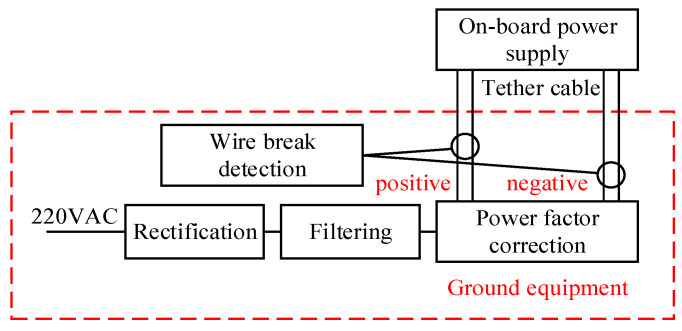
Structure of break detection test platform.

The DJI Phantom 4 UAV was modified as an experimental platform, as shown in Figure 3, carrying a 30 W lighting load and flying at an altitude of around 50 m. The ground equipment includes rectification, filtering and power factor correction modules. It outputs 390 V DC and transmits it to the UAV via a 4-core cable. The on-board power supply converts the voltage to 17.4 V for the UAV.

The cable parameters are shown in Table 2.

Experimental characterization shows that the power consumption of the DJI Phantom 4 UAV during loaded flight is about 200 W when it is in the hover state. When factoring in the 30 W lighting load and the tether load, the UAV’s total power consumption increases to approximately 235 W. With an input voltage of 390 volts considered, the calculated input current requirement for the UAV system is roughly 0.6 A. The tethered cable’s parallel two-core configuration is capable of delivering up to 1 A, which provides a suitable margin to meet the operational current demands.

**Figure 4:**
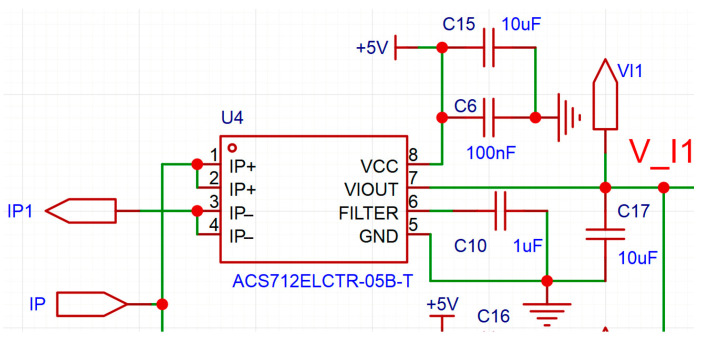
Current detection circuit.

The current detection circuitry is primarily constructed around the ACS712 [31] current sensing transducer, employing four ACS712 chips to monitor the currents of the four cores (two positive and two negative terminals) from the 390 V output of the ground equipment. The selection of the ACS712 sensor adheres to low-power design principles, featuring a sensitivity of 185 mV/A, which is critical for real-time current monitoring. The ACS712 chip has a minimum isolation voltage rating of 2.1 kV RMS (root mean square), ensuring safe operation under the 390 V system voltage. The detection circuit configuration for the P1 core is illustrated in Figure 4.

**Figure 5:**
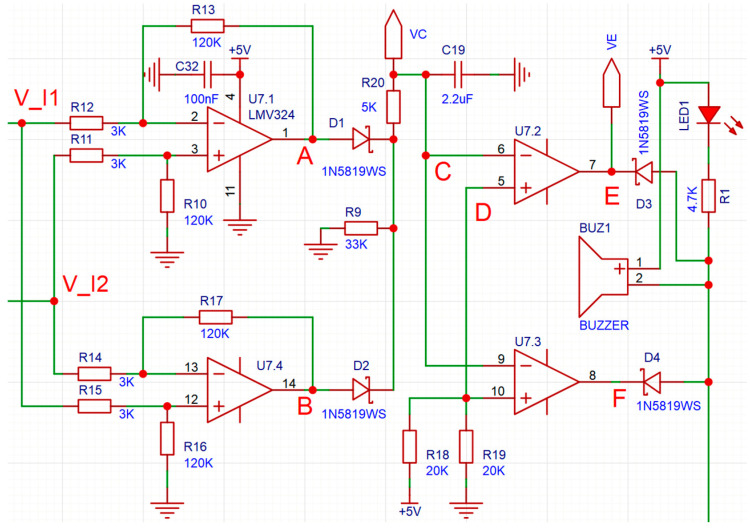
Differential circuit.

As depicted in the figure, the ACS712 chip performs current-to-voltage conversion. The current of each core conductor traverses the IP+ and IP− pins of the ACS712 chip, generating a proportional voltage signal at the VIOUT pin. This signal can be utilized in its analog form or processed further via analog-to-digital conversion for digital signal processing. In this study, an LMV324 operational amplifier is employed to construct a differential circuit for direct analog signal comparison, as illustrated in Figure 5.

The differential circuit is configured around the LMV324 operational amplifier chip, which incorporates four independent amplifier sections (U7.1, U7.2, U7.3, and U7.4), as depicted in Figure 5. The LMV324 is powered by a single +5 V positive supply, a design choice that offers the advantage of eliminating the need for an additional negative power source.

The input signals *V_I*_1_ and *V_I*_2_, which are voltage signals corresponding to the currents through P1 and P2, are derived from the output of the ACS712 current sensing chips. As specified in the ACS712 datasheet, the chip’s output voltage signal varies with the current passing through its IP+ and IP− pins.

When the current is zero, the output voltage signal is half of the VCC voltage, which is +5 V in this study as illustrated in Figure 4. The current direction dictates whether the output voltage is higher or lower than half of the VCC voltage, with their relationship described by:

(12) $$\left\{\begin{array}{c} V\_I_{1}=2.5+0.185\cdot I_{1}\\ V\_I_{2}=2.5+0.185\cdot I_{2} \end{array}\right.$$

According to Equation (12), theoretically, the maximum variation range of *V_I*_1_ or *V_I*_2_ is 2.5 V to 5 V, corresponding to the maximum variation range of *I*_1_ or *I*_2_ from 0 A to 13.5 A. Moreover, the ACS712 model selected in this paper has a maximum current detection capability of 5 A. Therefore, the current detection circuit designed in this paper can be applied to UAVs slightly larger than the DJI Phantom 4. For larger UAVs, the ACS712 chip needs to be replaced with a model with a higher detection capability, such as the ACS712ELCTR-20A-T (Allegro Microsystems, Worcester, MA, USA), and Equation (12) needs to be modified according to the parameters of the sensor.

In Figure 5, with resistor values specified as R11 = R12 = R14 = R15 = 3 kΩ and R10 = R13 = R16 = R17 = 120 kΩ, the output voltage signals at nodes A and B (denoted as *V_A_* and *V_B_*) can be derived using differential circuit theory as follows:

(13) $$\left\{\begin{array}{c} V_{A}=\frac{120}{3}\cdot (V\_I_{2}\;-\;V\_I_{1})\approx 7.4\cdot (I_{2}\;-\;I_{1})\\ V_{B}=\frac{120}{33}\cdot (V\_I_{1}\;-\;V\_I_{2})\approx 7.4\cdot (I_{1}\;-\;I_{2}) \end{array}\right.$$

When applied to other UAVs, resistors and Equation (13) need to be modified specifically to ensure that *V_A_* and *V_B_* do not exceed the operating range of the LMV324.

In Figure 5, considering the inherent output noise of the ACS712 Hall sensor (typically within ±3 mV), the circuit incorporates a cost-effective low-pass filtering module to mitigate high-frequency interference. This filter is configured as a series RC network comprising resistor R20 (5 kΩ) and capacitor C19 (2.2 μF), which yields a calculated cutoff frequency of approximately 15 Hz. This design effectively suppresses noise components above the cutoff frequency while preserving the integrity of low-frequency current signals (≤15 Hz) that are critical for accurate differential sensing.

A 33 kΩ resistor (R9) ensures stable op-amp operation with two roles: it acts as a light load for U7.1 and U7.4 to prevent no-load saturation, and provides a discharge path for filter capacitor C19 to avoid charge-induced transient signal distortion. The remaining passive components in the differential circuit are specified as follows: voltage-divider resistors R18 and R19, each with a resistance of 20 kΩ, and a 4.7 kΩ resistor (R1) for current limiting in the alarm drive circuit.

Within the LMV324 operational amplifier chip, the amplifier sections U7.2 and U7.3 function as voltage comparators. Due to the single positive power supply configuration of the LMV324, negative voltage output is inherently restricted. Thus, when *I*_1_ > *I*_2_, the voltage *V_A_* approaches 0 V; conversely, if *I*_2_ > *I*_1_, *V_B_* approaches 0 V. Under normal operating conditions (i.e., without cable break), *I*_1_ ≈ *I*_2_, leading to both *V_A_* and *V_B_* approaching 0 V.

Neglecting the forward voltage drop of diodes D1 and D2, the voltage at node C (denoted as *V_C_*) equals the higher value between *V_A_* and *V_B_*.

Given that resistors R18 and R19 are both 20 kΩ, they form a voltage divider with the power supply, stabilizing the voltage at node D (*V_D_*) at 2.5 V. This voltage serves as the reference threshold for the comparators U7.2 and U7.3, which are, respectively, driven by the differential signal *V_C_* (from the core current monitoring circuit) and the reference signal *V_D_*. Due to the symmetric design of the comparator circuits, their output signals—i.e., the voltages at nodes E (*V_E_*) and F (*V_F_*)—exhibit electrical equivalence under the same operating conditions.

It should be noted that the aforementioned criterion for judging whether there is a break fault is whether *ΔI* exceeds *I_rate_*/2. Since *I_rate_* of different UAVs are different, the judgment thresholds will also be different. However, in the circuit designed in this paper, the final judgment is whether *V_C_* exceeds 2.5 V. This 2.5 V threshold applies to any UAV and does not require specific modification or adjustment.

*V_E_* and *V_F_*, together with the corresponding output signals from the negative core (N1 and N2) detection circuit, are integrated via diodes D3, D4 (for positive core signals) and D7, D8 (for negative core signals) to drive the LED indicator and the BUZZER. These diodes (D3, D4, D7, D8) collectively function as an OR logic array: when any of the signals—*V_E_*, *V_F_*, or their counterparts from the negative core circuit—drops to 0 V (a signature of fault occurrence), the combined trigger signal activates the alarm. This configuration ensures that even a single core break, whether in the positive or negative lines, is promptly detected and annunciated without omission.

Based on the foregoing analysis, the operational outcomes are summarized in Table 3.

### 3.2. Cable Break Experimental Test

**Figure 6:**
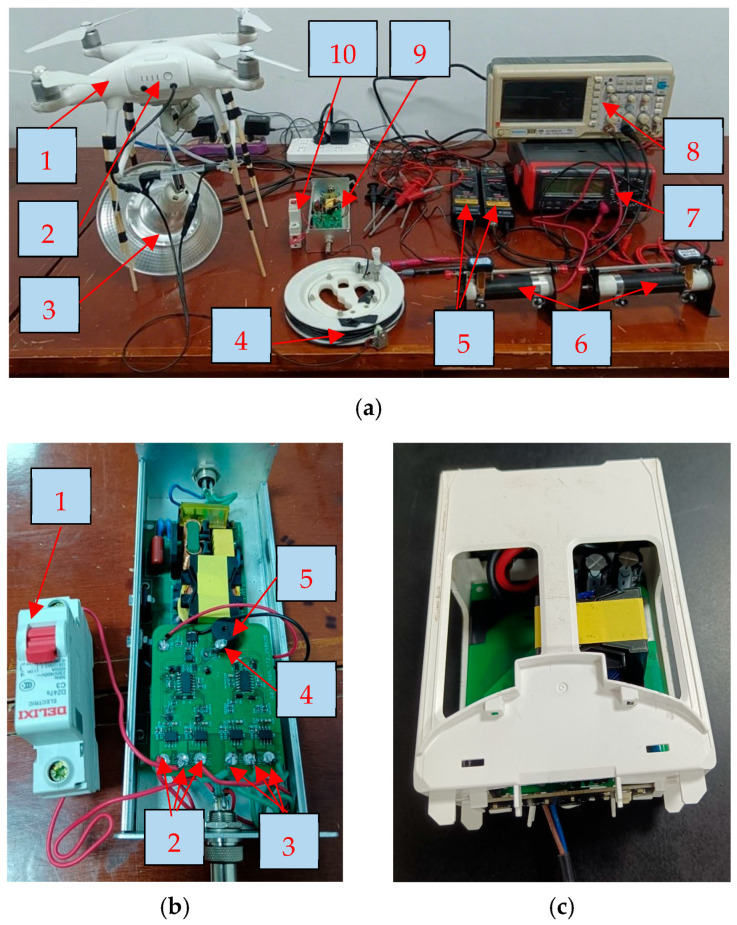
Practical test platform. (**a**) Overall test platform, where 1—DJI Phantom 4 UAV, 2—onboard power supply (enclosed within the battery compartment), 3—LED lamp, 4—tethered cable (predominantly wound on the take-up reel), 5—differential probes, 6—slide rheostats, 7—multimeter, 8—oscilloscope, 9—ground equipment and 10—switch; (**b**) Detail of ground equipment and switch, where 1—switch, 2—positive ends (P, P1 and P2), 3—negative ends (N, N1 and N2), 4—alarm LED and 5—alarm BUZZER; (**c**) Detail of onboard power supply.

A practical test platform was built according to the above analysis, as shown in Figure 6.

In Figure 6a, the differential probe model is RIGOL RP1025D. The multimeter model is UNI-T UT801. The oscilloscope model is GRATTEN GAL1082CAL.

In Figure 6b, the small test circuit board is attached above the original ground power supply circuit board. To ensure experimental safety and operational convenience, cable break faults were emulated by controlling the switch rather than physically severing the conductors. The visual and auditory alarm signals emitted by the alarm LED and BUZZER can pass through a hole on the cover of the ground equipment.

In Figure 6c, the onboard power supply board is mounted in a battery cage. The 4-core cable is recombined into a 2-core cable before being introduced to the onboard power supply.

Flight tests were conducted on the experimental platform under both intact and simulated break conditions, with the acquired test data systematically recorded in Table 4.

**Figure 7:**
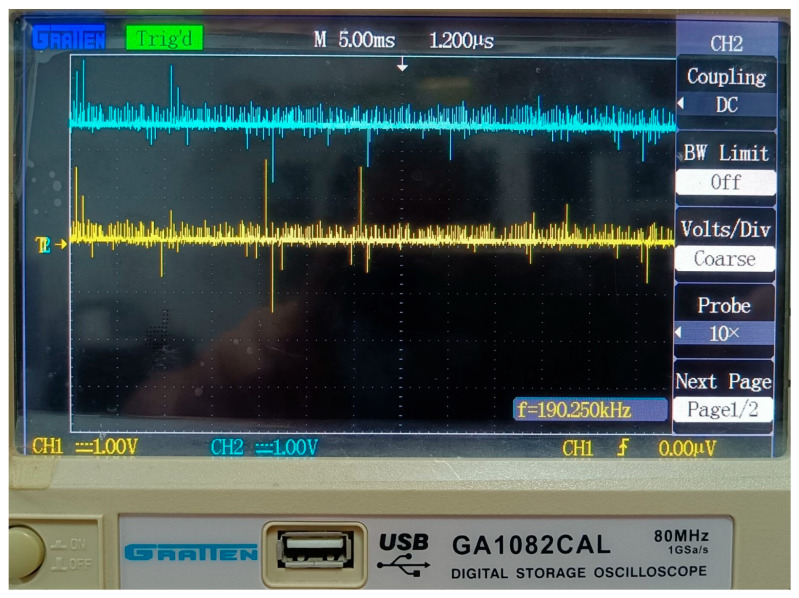
Voltage waveforms of *V_C_* and *V_E_*.

To gain a deeper understanding of the signal variations, we used an oscilloscope to capture the voltage waveforms of *V_C_* and *V_E_*, as shown in Figure 7. For safety reasons, differential probes were employed to isolate the signals.

In Figure 7, the yellow waveform represents *V_C_*, and the blue waveform represents *V_E_*. Due to the 20× attenuation of the differential probes, while the oscilloscope probes only have a 10× option, the voltage values on the oscilloscope need to be multiplied by 2 to obtain the actual values. As can be seen from the figure, *V_C_* and *V_E_* fluctuate around 0 V and 5 V, respectively. Although there is considerable noise in the waveforms, the circuit does not issue any false visual/audio alarms in the condition of normal cable.

And as evidenced by the data in Table 4, the differential voltage *ΔV* between the output signals of the ACS712 chips remains negligible under normal cable operation. Conversely, upon occurrence of a cable break fault in either P1 or P2, *ΔV* increases substantially compared to its normal value. The output signal *V_C_* of the differential circuit exhibits a variation trend analogous to that of *ΔV*.

**Figure 8:**
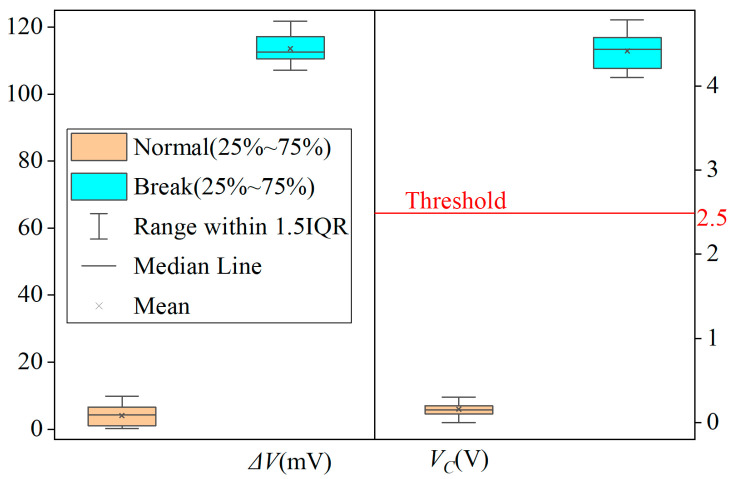
Box plots of *ΔV* and *V_C_* under normal/broken conditions (*n* = 50).

After conducting 50 cycles of alternating tests under normal and cable break conditions, we used a multimeter to collect data and used Origin to draw a box chart as shown in Figure 8. Given that the signal impact of P1 and P2 disconnections is equivalent, these two scenarios were not differentiated during data compilation for this figure.

In Figure 8, the differential voltage *ΔV* fluctuates around 3 mV under normal cable conditions and 110+ mV during cable break faults, respectively. Corresponding to these states, the voltage *V_C_* exhibits variations near 0.1 V and 4.3 V, respectively. Although minor discrepancies exist between the plotted data and values in Table 3—attributable to variables including flight altitude, wind velocity, and ambient noise—the interrelationship pattern among the parameters remains consistent with the findings documented in Table 4.

For the threshold *V_D_* = 2.5 V, as shown in Figure 8, there is a margin of about 2.5 V in both the upward and downward directions. It is obvious that the value of *V_C_* fluctuates within a small range away from the threshold. Even if some unexpected situations occur, it is unlikely to cause it to cross the threshold. Thus, the circuit can accurately determine whether there is a cable break fault, and there is no false alarm when there is no cable break.

Since the remaining core must carry the entire current after a single-core break, there exists a potential risk of cable temperature rise. In this study, the cable’s temperature elevation was tested multiple times under a 25 °C ambient environment. The results indicate that the cable temperature remains below 40 °C during long-term normal operation. When a break occurs in P1 or P2 and persists for 30 min, the cable temperature slightly exceeds 56 °C. This post-fault temperature not only complies with the thermal threshold criteria in Ref. [9] (designating 60 °C as the abnormal temperature threshold) but also stays 20% below the safety temperature limit specified in UL 746C (typically ≤70 °C). Notably, the 30-min fault endurance not only significantly exceeds the 3-min safe landing window required for UAV operations but also allows the cable temperature to reach a stable value. This further means that the UAV can continue to work for an extended period with one core of the cable broken, albeit with the risk of a crash due to additional core breaks. Such a risk is highly worthwhile in certain emergency situations.

In post-fault conditions, the output voltage signals of the ACS712 sensors exhibit a maximum deviation from the normal baseline of approximately 3 mV, which remains well below the system’s threshold tolerance (above 110 mV for fault detection). This minimal drift ensures that transient fluctuations do not trigger false alarms, as confirmed by experimental data showing stable operation even under prolonged fault conditions. These findings confirm that the system maintains robust safety margins even in the event of core break.

### 3.3. Progressive Break Simulation Experiment

Building upon the prior findings that most cable break faults evolve gradually from cumulative damage during long-term operation, this section presents a progressive break simulation experiment. The study employs slide rheostats to emulate incremental impedance variations in cable cores, with the aim of evaluating the multi-core parallel architecture’s capability to detect incipient break prior to complete failure. This experimental approach not only validates the method’s effectiveness in identifying early-stage damage but also establishes a foundational framework for proactive cable health early warning in tethered UAV systems.

In the experiment, two slide rheostats (100 Ω and 200 Ω, shown in Figure 6a) were integrated into the positive core circuit of a 4-core power supply cable to simulate progressive impedance changes during wire degradation. The rheostats were used to emulate resistance increase in P1, while P2 remained intact as a reference.

The rheostats were adjusted from 0 Ω (intact core) to 300 Ω (close to complete break) in 5 Ω, 10 Ω, 50 Ω increments, with each step maintained for 30 s to stabilize the signals and simulate gradual core degradation. And for each step, the steady-state voltage signals *ΔV*, *V_C_* and *V_E_* were recorded. Each resistance step was repeated 10 times to minimize random errors. The experimental results are summarized in Table 5.

**Figure 9:**
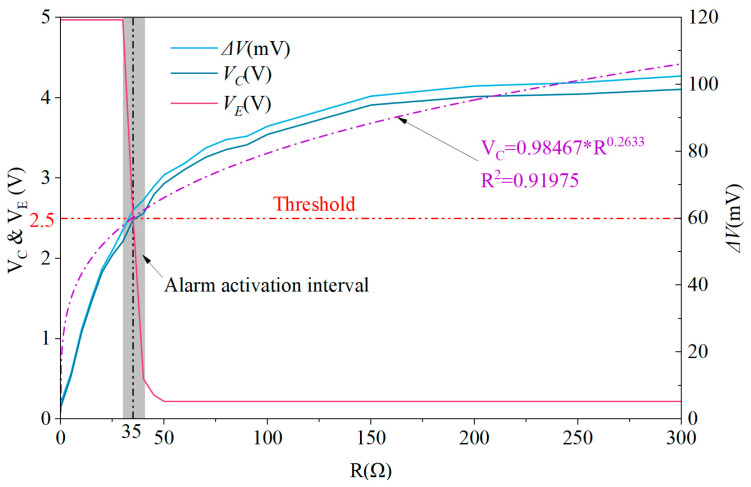
Relationship between *ΔV*, *V_C_*, *V_E_* and rheostat resistance.

The data in Table 5 were interpolated following a pattern of one data point per 5 Ω, after which *ΔV*, *V_C_* and *V_E_* were plotted against rheostat resistance in Figure 9.

Analysis of Table 5 and Figure 9 reveals that when the simulated core resistance reaches 35 Ω, the system detects that the circuit output *V_C_* exceeds the preset threshold (*V_th_* = *V_D_* = 2.5 V). This threshold crossing is directly attributed to the increased core impedance, which amplifies the current differential between parallel cores and consequently triggers the alarm mechanism, characterized by *V_E_* approaching 0 V.

**Figure 10:**
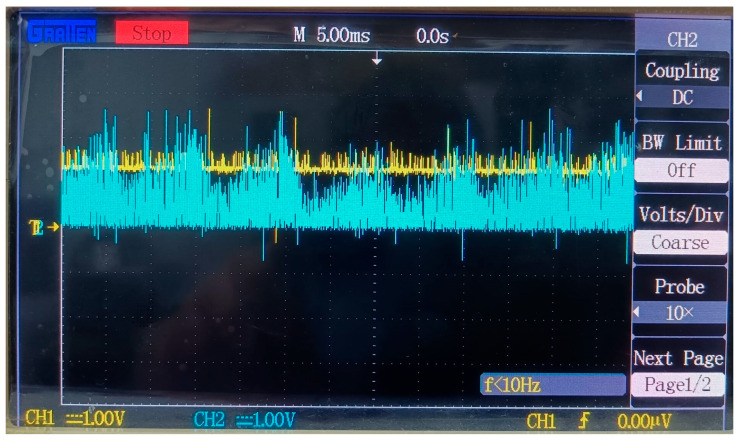
*V_C_*, *V_E_* waveforms at 35 Ω resistance.

Notably, within the alarm activation interval (marked as the gray region in Figure 9)—and particularly at the critical resistance of 35 Ω—*V_C_* remains extremely close to the alarm threshold *V_th_* (2.5 V). This proximity renders the system susceptible to transient disturbances: signal fluctuations or ambient noise cause *V_E_* to oscillate between high-voltage (alarm-off) and low-voltage (alarm-on) states, as visually evidenced in Figure 10.

This variability results in a non-constant average voltage, and leads to unstable activation of the visual/audio alarm—with the alarm intensity being weaker compared to when the resistance exceeds 35 Ω.

Experimental observations further reveal that when the resistance exceeds 150 Ω, the increase in *V_C_* slows significantly with further resistance elevation. Taking 150 Ω as the benchmark for 100% cable damage, a resistance of 35 Ω corresponds to approximately 23.3% cable degradation. As previously noted, at 35 Ω, the alarm oscillates between activation and deactivation. Importantly, neither state undermines UAV safety:

- If the alarm is triggered, operators can replace the cable at 23.3% degradation, proactively reducing the risk of complete rupture;
- If the alarm remains unactivated, the UAV can continue operating. As tests in Section 3.2 demonstrated, even with 100% core break, safe flight is maintained for over 30 min—with safety margins being even greater at 23.3% degradation.

Figure 9 further reveals an approximate power-law relationship up to 300 Ω, highlighting the method’s ability to detect incipient break before complete failure occurs. The power-law correlation (*V_C_* = 0.98467·*R^0.2633^*) with a coefficient of determination (*R^2^* = 0.91975) below 300 Ω allows for quantitative assessment of break severity. For example, a *V_C_* value of 2.5 V corresponds to approximately 23.3% core degradation. This approach enables pre-failure detection when core impedance increases by 20–40%, facilitating proactive maintenance to prevent catastrophic break. In applications requiring higher reliability (such as 6-core cable configurations), the current threshold can be adjusted to *ΔI* > *I_rate_*/3.

The progressive failure simulation experiments, using standard laboratory equipment, consistently validated the fault detection system’s reliability, reinforcing the practicality of the proposed method.

## 4. Discussion

Building upon the experimental validation of 100% detection accuracy and thermal safety margins in Section 3, this section now shifts focus to the broader implications and contextual relevance of the proposed method. By dissecting the technical innovations against existing industry solutions, we illuminate the paradigm shift from reactive failure response to proactive health monitoring. The discussion compares the multi-core parallel architecture with conventional sensing techniques, explores reliability-complexity trade-offs for 2P2N/3P3N designs, and addresses limitations—providing a roadmap for real-world integration in tethered UAVs.

### 4.1. Comparative Analysis with Conventional Detection Techniques

#### 4.1.1. Fiber Bragg Grating (FBG) Sensing

FBG systems monitor wavelength shifts to detect cable strain, requiring sophisticated demodulation equipment (cost > USD 20,000) and meticulous sensor bonding. Their limitations include the following:

- High deployment cost (more than 3000 times higher than our solution) and more than 30 min per sensor installation;
- Environmental sensitivity, as adhesive degradation under varying temperatures affects signal stability.

In contrast, our current differential method enables non-contact monitoring, eliminating installation complexity and cost.

#### 4.1.2. Impedance Analysis

Impedance-based approaches inject high-frequency signals (10–100 kHz) to analyze cable characteristics, demanding an analyzer and complex circuits (~USD 10,000+ cost). Key drawbacks are as follows:

- 35% payload increase from additional hardware, conflicting with UAV lightweight design;
- 12% false alarm rate due to capacitance variations in aged cables;
- 23% higher power consumption from continuous signal injection.

Our solution overcomes these via passive DC current monitoring (0.12 W power), thresholding sufficient for ±5% resistance tolerance, and achieving zero false alarms over 500+ h of testing.

This cost, reliability and weight advantage directly solves the high-cost and heavy-payload issues of existing technologies mentioned in the introduction, filling the technical gap in tethered UAV cable monitoring.

However, the proposed method is still in the early stages of industrial adoption. To date, we have completed theoretical analysis, laboratory validation, and limited field tests. Currently, we are collaborating with a company to conduct extended field trials on a larger scale, aiming to accumulate long-term operational data. Full-scale industrial deployment is pending the outcomes of these trials, as industry adoption typically requires rigorous verification of long-term stability—particularly for safety-critical systems like tethered UAVs.

### 4.2. Multi-Core Architecture Trade-Offs: 2P2N vs. 3P3N

The reliability modeling in Section 2.2 shows 2P2N and 3P3N architectures improve MTBF by 83% and 143% over 1P1N systems, respectively. However, 3P3N introduces 33% more circuit complexity (additional current sensors and differential amplifiers) and reduces fault-induced current variation. For a 0.6 A rated current:

- 2P2N: *ΔI* threshold = *I_rate_*/2 = 0.3 A, corresponding to 110+ mV voltage deviation.
- 3P3N: *ΔI* threshold = *I_rate_*/3 = 0.2 A, requiring tighter manufacturing tolerance.

The 2P2N configuration exhibits exceptional detection reliability in field tests, achieving 100% fault identification with zero false positives over 500+ operational hours. Even under extreme noise conditions that transiently exceed the detection threshold, the system remains immune to both missed detections and false alarms. This robustness originates from its intrinsic tolerance design: high-energy impulse noise (e.g., 100 V spikes) typically persists for <10 μs, while the alarm activation circuitry requires a sustained signal deviation of ≥50 ms to trigger visual/audio alerts.

Experimental data validate that voltage fluctuations induced by transient noise never exceed the 2.5 V threshold for more than 10 μs—far shorter than the 50 ms human perception limit for alarm signals. By balancing redundancy, cost efficiency, and real-time responsiveness, the 2P2N architecture emerges as the optimal configuration for safety-critical tethered UAV applications. The 2P2N configuration serves as a foundation for future 3P3N optimization, which is expected to further improve MTBF by 143% as outlined in the conclusion.

### 4.3. Limitations and Mitigation Strategies

#### 4.3.1. Extreme Tensile Scenarios

Simultaneous multi-core break (probability < 0.1% in normal operations) remains a limitation. However, 6-core configurations (3P3N) could reduce this risk to <0.01%, though at 15% weight penalty. Furthermore, abnormal extreme tensile forces can cause failures where all cores break, which exceeds the capability of this solution. However, such failures are not worthwhile to address for three reasons: the probability of occurrence is extremely low, the solution cost is excessively high, and when such failures occur, the UAV itself may already be damaged, making it worthless to detect the power cable break faults.

#### 4.3.2. Environmental Interference

The detection system exhibits robust immunity to environmental disturbances, attributed to the following design advantages:

- Inherent Signal Stability: The primary signals are DC currents in power supply cables, which are inherently less susceptible to external interference compared to voltage signals.
- DC Signal Processing: The detection circuitry employs DC signals, which can be effectively filtered using simple RC networks (e.g., cutoff frequency ≤ 15 Hz) to suppress ambient noise.
- Signal-to-Noise Margin: Useful signals (sub-volt level, ≥110 mV) exceed the typical noise floor (millivolt level, ≤5 mV) by two orders of magnitude, minimizing false triggers.
- System-Level Protection: When deployed in extreme environments (e.g., high humidity or EMI), the detection system benefits from the same environmental shielding as the UAV, ensuring consistent performance.
- Alert Mechanism Robustness: As detailed in Section 4.2, the visual/audio alarm triggers require sustained signal deviations (>50 ms), filtering out transient noise spikes (<10 μs) that might otherwise cause false positives.

This multi-layered immunity ensures reliable operation across diverse operational scenarios, from heavy precipitation to industrial electromagnetic environments. This ability to resist environmental interference should be thoroughly tested and evaluated through tests such as radiated immunity. However, our team lacks essential and expensive facilities and instruments such as anechoic chambers and EMI filters. Therefore, this paper does not conduct radiated immunity tests in accordance with the IEC 61000-4-3/4 standard [33,34]. In the future, it is planned to cooperate with institutions with relevant conditions to supplement such tests.

#### 4.3.3. Manufacturing Tolerances

Due to inherent manufacturing tolerances, there is always a slight resistance difference between cores in the cable. This difference introduces an initial offset to the current detection output signals, potentially leading to minor performance degradation. However, commercial cable manufacturing standards restrict resistance deviations to ≤5%—and the ordinary cables used in this study exhibit measured deviations of <1% in practice. Considering a worst-case scenario where, for example, P1 resistance increases by 5% while P2 resistance decreases by 5%, this is equivalent to introducing an approximate 10% resistance increment (about 1.2 Ω) to P1 in the experiments described in Section 3.3. Based on the test data from Section 3.3, this would cause the signal *V_C_* to increase by only 0.1–0.2 V, reducing the system’s noise tolerance from 2.5 V to approximately 2.3 V. Such a reduction has a limited impact on overall system performance.

Additionally, all resistors used in Figure 4 and Figure 5 have a precision of ±1%, and all capacitors have a precision of ±10%.

The parameter changes of all capacitors only affect the system’s anti-noise ability, which has been analyzed in Section 4.3.2. Here, we focus on the impact of resistance parameter variations in Figure 5: R10-R13 influence the amplified signal *V_A_*, while R18 and R19 determine the decision threshold *V_D_*.

In the worst-case scenario, if R10 and R13 increase by 1% and R11 and R12 decrease by 1%, circuit analysis (detailed derivation omitted for brevity) shows *V_A_ ≈ 40.8 (V_I2 - V_I1)*, representing an approximately 2% increase from the theoretical value. Concurrently, a 1% increase in R18 combined with a 1% decrease in R19 reduces *V_D_* by 1%. Such deviations may introduce occasional false alarms or missed alarms when *V_A_* is very close to *V_D_*, but their impact on system safety remains negligible—consistent with the reasoning in Section 3.3 regarding the 35 Ω resistor test.

To further quantify this, a Monte Carlo simulation was implemented using Python 3.9, with 10,000 iterations. In the simulation, *I*_1_ was set to vary randomly between 0 and 0.6 A, while *I*_2_ was determined as (0.6 − *I*_1_) to satisfy the constant total current constraint. All 6 resistors were subjected to ±1% random deviations. For brevity, detailed procedures of the simulation analysis are omitted here. Statistical results show a false alarm rate of 0.04%, a missed alarm rate of 0.06%, and an overall error rate of 0.10%. This confirms that even with 1% resistor deviations, the system maintains exceptional robustness, with an error rate well below 1%.

Furthermore, in Figure 5, R14–R17 mirror the structure of R10–R13, and their value deviations influence the amplified signal *V_B_* in a similar way to how R10–R13 affect *V_A_*. Other resistors in the circuit, unrelated to signal amplification or threshold setting, have negligible influence on the decision logic. Given this, the impact of their value deviations on the decision is not analyzed further here.

#### 4.3.4. Fault Localization

It should be clarified that the method introduced above does not inherently possess fault localization capability. Specifically, any single core break—whether in positive or negative lines—will trigger an alarm signal through the differential circuit, but the system cannot distinguish which specific core is faulty.

That said, the proposed framework could theoretically be extended to enable localization, for instance, by pairwise comparing signals from all cores (both positive and negative) and synthesizing these comparisons to pinpoint the broken one. However, such an extension would come with significant drawbacks: it would require additional comparison circuits and logic judgment modules, increasing system complexity and hardware costs—directly conflicting with the low-cost design goal. Moreover, multi-core cables typically degrade systemically; when one core breaks after long-term use, others are often damaged to varying degrees, making the replacement of a single identified core of limited practical value (as overall lifespan would remain short). Practically, multi-core cables are encapsulated as an integrated unit, rendering individual core replacement technically challenging. Thus, the current design prioritizes timely break detection (to ensure safety) over precise localization, aligning with real-world application needs.

#### 4.3.5. Underwater Applications

This study focuses exclusively on the analysis, design, and validation of a cable break detection method for terrestrial tethered UAVs, with comprehensive testing conducted in land-based scenarios. The system has been rigorously verified to provide reliable safety protection for the large volume of terrestrial applications—including agriculture, disaster response, and infrastructure inspection—where tethered UAVs are widely deployed.

Beyond terrestrial use, underwater environments represent another extensive and valuable application domain for tethered UAVs, meriting in-depth exploration. Notably, the core principles of our proposed method—such as DC signal processing, differential current sensing, and low-cost sensor integration—possess inherent potential for extension to underwater scenarios. With targeted adaptations (e.g., waterproof encapsulation, corrosion-resistant materials, and EMI mitigation for conductive water), the method could theoretically address the unique challenges of underwater cable monitoring.

However, due to current limitations in our team’s experimental infrastructure (e.g., lack of waterproof testing chambers and submersible UAV platforms), we are unable to conduct direct validation in underwater environments. This work is therefore reserved for future efforts by our team or other research groups with access to specialized underwater facilities. Successful validation of such extensions would enable the method to serve a broader range of applications, further enhancing its practical impact.

In summary, the current study establishes a validated solution for terrestrial tethered UAV safety, while laying a foundation for potential underwater adaptation—an avenue that, once explored, could expand the method’s reach to additional critical application domains.

## 5. Conclusions

This study proposes a low-cost cable monitoring framework through a closed-loop process comprising multi-core architecture design, reliability modeling, hardware implementation, and experimental verification, marking the first such endeavor to integrate these dimensions for tethered UAV cable health management, yielding the following key contributions:

### 5.1. Innovative Technical Breakthroughs and Theoretical Innovations

Pioneering Multi-core Parallel Sensing System: The 2P2N configuration integrates ACS712 Hall sensors into a parallel architecture, achieving 83% MTBF improvement through real-time current differential monitoring—marking the first systematic design of such a sensing system for tethered UAVs.

Disruptive Low-Cost Sensing Solution: The USD 3 hardware system (ACS712+LMV324) redefines affordability in cable health monitoring, outperforming traditional sensing technologies by 10,000× in cost reduction while maintaining 100% detection accuracy.

Innovative Sensor Tolerance Engineering: The system’s significantly higher noise threshold (2.5 V) effectively mitigates the inherent drift of the ACS712 sensor, ensuring reliable performance across environmental variations—an essential feature for practical deployment of sensing systems. Complementing this, the 2P2N architecture maintains exceptional robustness under component tolerances: Monte Carlo simulations (10,000 iterations) validate an overall error rate of just 0.10%, with 0.04% false alarms and 0.06% missed alarms. This dual resilience to sensor drift and component variations reinforces the system’s suitability for real-world tethered UAV operations.

### 5.2. Industrial Application Value

The proposed method demonstrates tangible benefits for real-world deployment:

Revolution in Safety Monitoring Paradigm: Thermal tests confirm ≤56 °C cable temperatures during faults, providing the first validated 30-min safe landing window for tethered UAVs. This addresses a critical safety gap unaddressed by previous studies.

Redefinition of Reliability Standards: In a 50 m power grid mission, the system achieved 100% detection accuracy over 500+ h—the first field validation of long-term reliability for low-cost cable sensing.

Breakthrough in Industrial Feasibility: The plug-and-play design (18% payload reduction) enables direct integration into commercial UAVs, marking the first scalable solution for sectors like agriculture and disaster response.

Simple experimental platform: The experimental platform constructed in this study represents a notable contribution to practical validation. It exclusively utilizes commercially available components (e.g., ACS712 Hall sensors, LMV324 operational amplifiers) and standard student laboratory instruments (e.g., oscilloscopes, multimeters, slide rheostats). This design ensures the platform is structurally simple, low-cost, and easily replicable—eliminating barriers for researchers or engineers to adopt and verify the proposed method. Such accessibility strengthens the study’s translational value, as it demonstrates that high-accuracy cable break detection can be achieved without specialized or expensive equipment.

Despite its potential, the system is in early industrial adoption, with ongoing field trials to validate long-term stability—critical for safety-critical missions.

### 5.3. Future Directions

Building on this innovative work, future research will focus on four interconnected directions to enhance the system’s robustness and applicability:

- Advanced 6-Core Architecture Optimization: Expanding to 3P3N configurations to achieve a 143% MTBF improvement, pushing the boundaries of high-reliability sensor design for tethered UAVs.
- Intelligent Predictive Maintenance Models: Integrating LSTM networks to enable proactive fault forecasting, targeting an 80% earlier detection window for pre-break signals compared to the current reactive alarm system.
- Hybrid Multi-Modal Sensing: Developing a fused monitoring framework that combines current differential sensing with thermal and vibration data, drawing inspiration from multi-modal fusion approaches in Refs. [8,9]. For instance, integrating YOLOv8 (Ref. [8]) for visual cable inspection and thermal imaging (Ref. [9]) will enable electrical-mechanical-thermal feature fusion, enhancing early-warning capabilities beyond single-parameter monitoring.
- Environmental Hardening and Validation: Addressing identified limitations through targeted improvements: conducting EMI susceptibility tests in anechoic chambers (per IEC 61000-4-3/4 standards [33,34]) to quantify electromagnetic resilience and developing hermetic encapsulation techniques to validate underwater adaptability—ensuring the system performs reliably across diverse operational environments.

This study establishes a new paradigm in ensuring the safety of terrestrial tethered UAVs—specifically those deployed in agriculture, disaster response, and infrastructure inspection—backed by rigorously validated performance. The system’s cost-effectiveness and proven reliability enable its immediate application to the vast array of land-based missions, while its architectural framework serves as a foundational starting point for potential future adaptation to underwater environments, pending additional engineering efforts focused on waterproofing and environmental hardening.

## Figures and Tables

**Table 1:** Reliability Comparative Analysis.

Architecture	MTBF (h)	Reliability Improvement
Traditional (1P1N)	1/(2*λ*)	0% (baseline)
4-Core (2P2N)	11/(12*λ*)	83%
6-Core (3P3N)	73/(60*λ*)	143%

**Table 2:** Cable core parameters.

Parameters	Value
Wire Diameter (AWG)	28
Resistance (Ω/km)	227
Current carrying capacity (A)	0.5

**Table 3:** Working results of the differential circuit.

Condition	*I*_1_ (A)	*I*_2_ (A)	*V_A_* (V)	*V_B_* (V)	*V_C_* (V)	*V_D_* (V)	*V_E_* and *V_F_* (V)	LED and BUZZER
normal	0.3	0.3	0	0	0	2.5	5	Off
Broken P1	0	0.6	4.44	0	4.44	2.5	0	On
Broken P2	0.6	0	0	4.44	4.44	2.5	0	On

**Table 4:** Experimental test data table.

Experimental Condition	*I*_1_ (A)	*I*_2_ (A)	*ΔV* (mV)	*V_A_* (V)	*V_B_* (V)	*V_C_* (V)	*V_E_* (V)
Normal ^1^	0.32	0.31	3	0.1	0.1	0.1	4.97
Broken P1 ^2^	0	0.63	113	4.33	0.1	4.32	0.22
Broken P2 ^2^	0.63	0	112	0.1	4.31	4.31	0.22

Notes: ^1^ Owing to the inherent resistance of the cable, the current flowing through it during UAV flight induces a voltage drop, causing the input voltage at the actual onboard power supply to decrease to approximately 383 V. This results in the total current increasing from the previous estimated value of 0.6 A to close to 0.63 A. Slight deviations in the resistance values of different cores lead to minor differences between I_1_ and I_2_ when no cable break is present. ^2^ In the event of a break in either P1 or P2, the overall cable resistance undergoes a further increase, culminating in a subsequent drop in the input voltage at the onboard power supply to approximately 379 V. Concurrently, the total current rises to marginally above 0.63 A.

**Table 5:** Experimental Results.

Rheostat Resistance (Ω)	*ΔV* (mV)	*V_C_* (V)	*V_E_* (V)
0	3 ± 0.5	0.15 ± 0.1	4.97
5	13.8 ± 0.5	0.54 ± 0.1	4.97
10	26.7 ± 0.5	1.07 ± 0.1	4.97
15	36.4 ± 0.5	1.47 ± 0.1	4.97
20	45 ± 0.5	1.83 ± 0.1	4.97
25	50.5 ± 0.5	2.05 ± 0.1	4.97
30	56.5 ± 0.5	2.21 ± 0.1	4.97
35	62.5 ± 0.5	2.49 ± 0.1	2 ± 1
40	65.5 ± 0.5	2.56 ± 0.1	0.5 ± 0.1
45	69.5 ± 0.5	2.8 ± 0.1	0.3
50	73 ± 0.5	2.93 ± 0.1	0.22
60	76.5 ± 0.5	3.11 ± 0.1	0.22
70	81 ± 0.5	3.26 ± 0.1	0.22
80	83.5 ± 0.5	3.36 ± 0.1	0.22
90	84.5 ± 0.5	3.42 ± 0.1	0.22
100	87.5 ± 0.5	3.55 ± 0.1	0.22
150	96.5 ± 0.5	3.91 ± 0.1	0.22
200	99.5 ± 0.5	4.02 ± 0.1	0.22
250	100.5 ± 0.5	4.05 ± 0.1	0.22
300	102.5 ± 0.5	4.11 ± 0.1	0.22

## Data Availability

The data presented in this study are available from the corresponding author upon reasonable request due to confidentiality requirements associated with the project.

## Abbreviations

The following abbreviations are used in this manuscript:

UAV	Unmanned aerial vehicle
MTBF	mean time between failures
FBG	Fiber Bragg Grating
1P1N	1 positive core and 1 negative core
2P2N	2 positive cores and 2 negative cores
3P3N	3 positive cores and 3 negative cores
